# Case report: Hepatic epithelioid angiomyolipoma with elevated alpha-fetoprotein and a history of breast cancer

**DOI:** 10.3389/fsurg.2022.991228

**Published:** 2022-09-09

**Authors:** Xiaowei Zhang, Jun Chen, Bifei Huang, Lihong Wang

**Affiliations:** ^1^ Department of Pathology, Affiliated Dongyang Hospital of Wenzhou Medical University, Dongyang, China; ^2^ Department of Nuclear Medicine, Affiliated Dongyang Hospital of Wenzhou Medical University, Dongyang, China; ^3^ Department of Orthopaedic Surgery, Affiliated Dongyang Hospital of Wenzhou Medical University, Dongyang, China

**Keywords:** hepatocellular carcinoma, hepatic epithelioid angiomyolipoma, alpha-Fetoprotein, breast cancer, case report

## Abstract

**Background:**

Perivascular epithelioid cell tumors have characteristic histological and immunohistochemical profiles. Epithelioid angiomyolipomas in the liver have particularly rare characteristics. These tumors are relatively small and lack clinical and imaging specificity. Thus, they can be easily misdiagnosed as other primary or metastatic tumors prior to surgery. Due to the significant epithelioid morphology and atypia of hepatic epithelioid angiomyolipoma (HEAML), intraoperative frozen section pathologic diagnosis might be challenging.

**Case presentation:**

A 33-year-old woman was admitted to our hospital for elevated alpha-fetoprotein (AFP) levels after a regular checkup following breast cancer surgery. Initially, liver cancer was suspected based on enhanced magnetic resonance imaging and color Doppler ultrasonography. Her serum AFP levels were 23.05 ng/ml. The patient underwent laparoscopic right hepatic tumor resection. Based on intraoperative cryopathology, hepatocellular carcinoma was considered a likely diagnosis. However, postoperative pathology confirmed a right HEAML. The patient underwent regular checkups for 23 months without exhibiting recurrence or distant metastasis.

**Conclusion:**

HEAML can be easily misdiagnosed on preoperative imaging and intraoperative cryopathology. Medical professionals must be aware of this possibility and proceed with caution. Postoperative pathological examination with assessment of IHC markers was helpful in diagnosing HEAML. HEAML has low malignant potential. Surgical resection is the mainstay of treatment, and most patients have a good prognosis.

## 1. Introduction

Hepatic epithelioid angiomyolipoma (HEAML) is a rare neoplasm and is an unusual type of the perivascular epithelioid cell tumor. On clinical and imaging examination, HEAML is often misdiagnosed as liver cancer or other types of lesions. Therefore, it is treated with mass complete resection. On pathological examination, various morphologies of HEAML resemble other benign and malignant liver tumors. Herein, we present a case of HEAML in a patient with a history of breast cancer and elevated alpha-fetoprotein (AFP) levels. To the best of our knowledge, this is the first case of HEAML presenting with elevated AFP levels. Our study describes the histopathological and immunohistochemical characteristics of the HEAML tumor and clinical and imaging manifestations of the case. The patient had no history of hepatitis or cirrhosis.

## 2. Case description

A 33-year-old woman was admitted to our hospital on January 16, 2019, for elevated AFP levels. During a routine follow-up after combination therapy for breast cancer, her serum test results showed AFP levels of 23.05 ng/ml (normal levels: <13.6 ng/ml). At that time, the patient had no abdominal pain or distension, anorexia, nausea, vomiting, acid regurgitation, yellow skin, or eyes, urine, or skin irritation, and she exhibited no features of chronic liver disease; meanwhile, there were no clinical features to suggest metastatic breast cancer. Physical examination revealed a 12 cm surgical incision due to a modified radical mastectomy of the left breast that the patient had undergone six years prior at our hospital. This surgery had been performed to treat the invasive carcinoma of her left breast after completing four cycles of postoperative chemotherapy. The extent of wound healing at the surgical site indicated no adverse events. Furthermore, no nodules were found. The liver and spleen were not swollen under the costal xiphoid process with no percussion pain in the liver area. Moreover, the abdomen was soft without tenderness.

### 2.1. Ethics declarations

This study was approved by the Dongyang Hospital, Affiliated to Wenzhou Medical University, Ethics Committee. The patient provided written informed consent to participate in this study. Additionally, written informed consent was obtained from the patient for the publication of any potentially identifiable images or data included in this article. This study was reported in agreement with principles of the CARE guidelines (1).

## 3. Diagnostic assessment

### 3.1. Imaging findings

**Figure 1:**
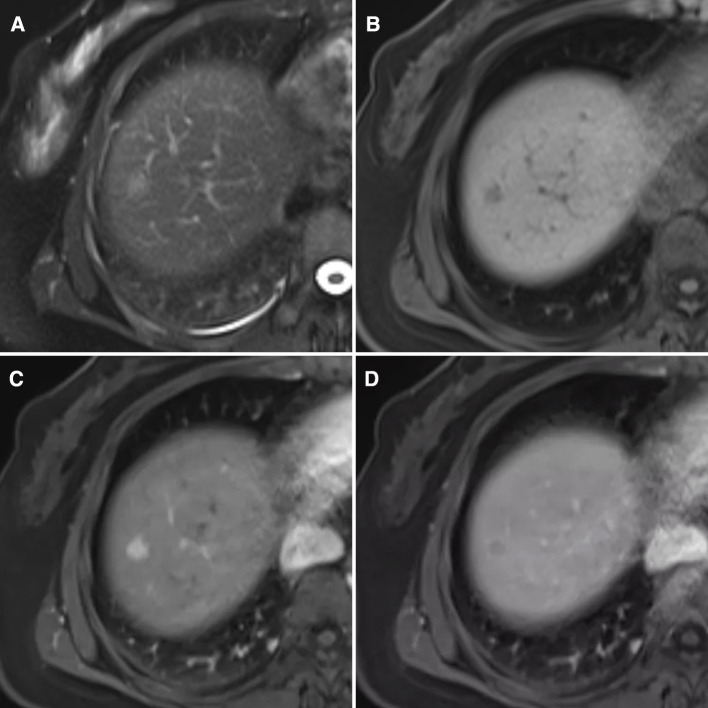
MRI findings of this case (**A**) heterogeneous hyperintensity on T2 lipid-pressing sequence is shown. (**B**) Low signal on T1 lipid-pressing sequence is shown. (**C**) The arterial phase is markedly enhanced. (**D**) Hypointensities in the portal phase are shown.

Contrast-enhanced magnetic resonance imaging (MRI) scans of the liver revealed abnormal findings, including low signals on T1-weighted imaging and slightly elevated signals on the T2 fat saturation sequence. An area of hypodensity was observed in the right anterior segment of the liver with a 1.0 cm diameter and a clear boundary. Afterward, there was a significant enhancement in the arterial phase and a decrease in the portal phase. There was no dilatation of the intrahepatic bile duct and no lesions were detected in the region of the hepatic hilum. In addition, no swollen lymph nodes were found in the retroperitoneal area. The lesion in the right anterior segment of the liver was suggestive of hepatocellular carcinoma (Figure 1).

#### 3.1.1. Contrast-enhanced liver ultrasound

**Figure 2:**
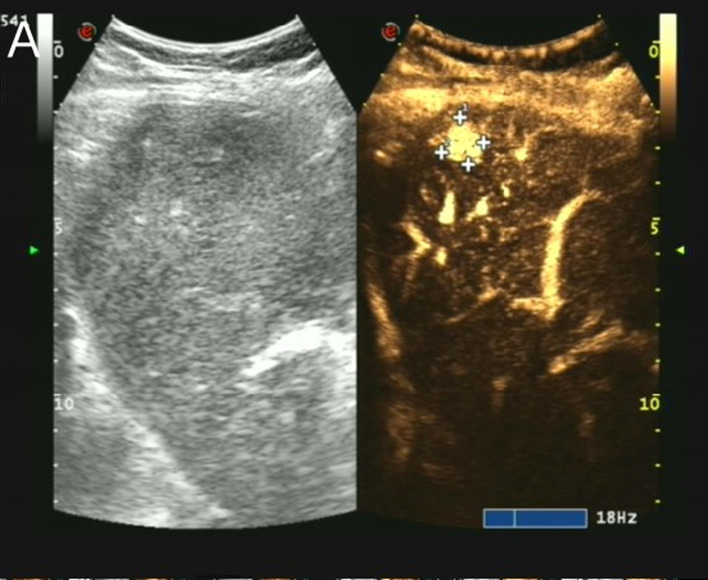
Contrast-enhanced liver ultrasound findings of this case contrast-enhanced ultrasonography of the nodules in the upper segment of the right anterior lobe of the liver showing washout performance (surrounded by crosses).

A slightly low echo area of approximately 13 × 11 mm in size was found in the upper segment of the right anterior lobe of the liver, with an irregular shape and uneven internal echo. The ulnar vein was injected at 10 minute intervals with 1.5 ml sulfur hexafluoride as ultrasound contrast agent. Contrast-enhanced ultrasonography revealed nodules in the upper segment of the right anterior lobe of the liver that began to strengthen at 12 s, reached a peak at 20 s, and then began to subside. This showed arterial hypervascularity with washout, with a range of approximately 13 × 13 mm, suggesting a high possibility of primary hepatic carcinoma (Figure 2).

### 3.2. Treatment

Laparoscopic-assisted resection of the right hepatic tumor was performed. During the operation, the tumor was found to be 1.0 cm in size, with clear edges, and >1 cm from the cut edge. The tumor was completely resected. In the frozen section pathological analysis of the right liver specimen, the possibility of hepatocellular carcinoma was tested first. The patient recovered and was discharged post-operatively.

### 3.3. Histopathology

#### 3.3.1. Postoperative pathology

##### 3.3.1.1. The right liver

**Figure 3:**
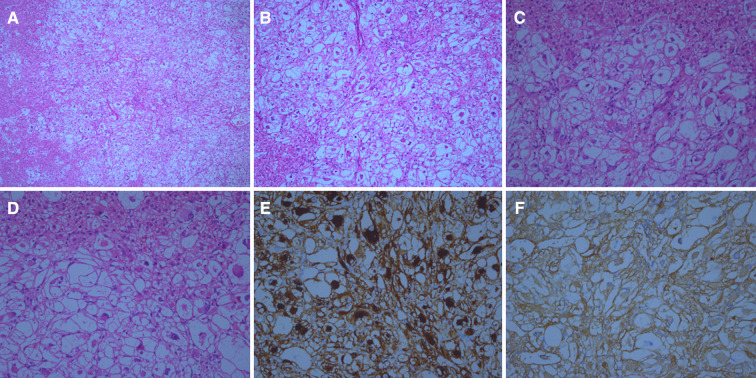
Histological findings of this case (**A–D**) hematoxylin and eosin staining of the tumor under multiple magnifications (**A**, ×50) (**B**, ×100). (**C**, ×200) (**D**, ×400). Microscopically, the tumor boundary is unclear and grows into the surrounding liver tissue. Epithelioid-like tumor cells can be seen arranged in a nest-like shape. Vascular components are visible, but adipocytes and smooth muscle components are not found. Under a high microscope objective, the tumor nuclei are round or oval with obvious nucleoli and atypia. (**E**) Tumor cells stained positive for HMB 45. Magnification at ×200. (**F**) Tumor cells stained positive for SMA. Magnification at ×200.

A 4.5 × 3.3 × 1.8 cm specimen of the liver tissue was retrieved during the operation, including a grayish-yellow mass (1.2 × 0.9 cm in size), which was soft and had a clear boundary. Light microscopy analysis showed that the liver tumor nodules were arranged by large polygonal epithelioid cells in the form of sheet nests or irregular beam cables, divided by thin-walled vessels of different sizes. The tumor cell cytoplasm was transparent, with vacuoles in the periphery. Eosinophilic granules could be seen in the cytoplasm. The nuclei were round or oval and large with clearly visible nucleoli and deep chromatin. The nodule boundaries were unclear as they grew into the surrounding liver tissue (Figures 3A–D).

##### 3.3.1.2. Pathological diagnosis

Epithelioid angiomyolipoma of the right liver (tumor size: 1.2 × 0.9 cm) with negative incisional margins.

##### 3.3.1.3. Immunohistochemical staining results

The tumor cells were positive for estrogen receptor (1 + 40%), progesterone receptor (2 + 10%), desmin (focal + ), E-cadherin, vimentin, and human melanoma black (HMB) 45, smooth muscle actin (SMA) (Figures 3E, F). The specimen was negative for GATA binding protein 3 (GATA3), CerbB-2, AFP, glypican-3, HepPar1, cytokeratin (CK) (AE1/AE3), cell adhesion molecule 5.2, CK20, CDX2, CK7, CK8, and S-100, with a Ki-67 index of 5%.

### 3.4. Postoperative follow-up

Over the 23 months of follow-up, no recurrence or distant metastasis was observed on computed tomography and MRI scans.

## 4. Discussion

Perivascular epithelioid cell tumors are a group of mesenchymal lesions that share similar vascular wall morphology and express melanin and smooth muscle markers. Epithelioid angiomyolipoma, which is classified as a perivascular epithelioid cell tumor, is especially unique and rare (2). In 1976, Ishak et al. first described HEAML as a rare mesenchymal tumor, mostly occurring in women, with approximately 5%–10% of these tumors being accompanied by nodular sclerosis (3). To date, the pathogenesis of HEAML is unclear. The possible etiologies of tumors include undifferentiated neural crest, differentiated smooth muscle cells, and perivascular cells. Studies have indicated that changes in the mammalian target of the mechanistic target of rapamycin (mTOR) pathway and loss of heterozygosity (LOH) of the tuberous sclerosis syndrome gene were the common pathogeneses of HEAML (4).

HEAML tends to occur in young and middle-aged women, with a male to female ratio of 1:5. Usually, these tumors are small and cause no characteristic clinical symptoms. However, when a tumor is large, the patient may experience abdominal distension and pain (5). Our patient had elevated AFP levels without experiencing any other symptoms suggestive of HEAML. The elevated AFP levels were coincidentally found during the patient's regular follow-up examination and imaging after being treated for breast cancer. Since HEAML contains only a small amount of fat or lacks it entirely, it is easily misdiagnosed for other malignant tumors during imaging examination before tumor resection (6). The preoperative liver MRI and color Doppler ultrasound in the present case suggested a possibility of liver cancer. Since the patient's AFP level was elevated, intraoperative frozen section analysis was needed to differentiate tumor type but this has the potential to be inconclusive. Thus, vigilance was needed to establish the correct diagnosis. Diagnosing HEAML by fine-needle puncture before surgery is necessary.

Although pathological examination is the gold standard for diagnosing HEAML, this patient had a history of breast cancer, and the tumor marker (AFP) was elevated, which could result in an incorrect pathological diagnosis. Microscopic analysis revealed that the HEAML cells were round or polygonal. Their cytoplasm was rich, slightly acidophilic or transparent, and sometimes contained fat vacuoles. The nuclei were median or partial, large, round or ovoid, with obvious nucleoli and rare mitotic images (7). IHC revealed that HEAML tissue was positive for melanocytic markers (HMB 45 and Melan-A) and smooth muscle cell markers (SMA and desmin) (8). Although transcription factor E3 can be expressed in some cases, there are usually no related gene expression changes. HEAML tissue does not express epithelial markers, S-100, SOX11, CD31, and CD34.

The morphology of HEAML could can be diverse making it is easy to confuse this tumor with other primary or metastatic tumors in the liver. A comprehensive IHC evaluation is required to distinguish HEAML diagnosis from other primary and metastatic liver tumors. Key ways to differentiate HEAML from other hepatic primary or metastatic tumors include the following:

First, in comparison to metastatic breast cancer, it usually presents a nest-like or glandular structure and IHC expression of markers (GATA3 and GCDFP15). Our patient had a history of breast cancer that needed to be excluded. It can sometimes be difficult to identify HEAML by evaluation using frozen section biopsy.

Second, when considering hepatocellular carcinoma, due to the significant epithelioid morphology and atypia of HEAML, it can easily be misdiagnosed as hepatocellular carcinoma, particularly based on intraoperative cryopathology diagnosis. In the present case, cryopathology analysis incorrectly identified HEAML as hepatocellular carcinoma. HEAML tissue expresses melanocytic markers, not epithelial markers. In addition, patients with hepatocellular carcinoma usually have a history of hepatitis and cirrhosis. Thus, it is easy to distinguish between the two after comprehensive analysis of postoperative pathology.

Third, HEAML demonstrates melanocyte differentiation and cell atypia, which is easily misdiagnosed as malignant melanoma. However, malignant melanoma usually expresses S-100 protein and is negative for smooth muscle cell markers. Based on patient history and IHC, malignant melanoma can be differentiated from HEAML.

Surgical resection is the preferred treatment of HEAML and should be performed as early as possible to avoid gradual growth and rupture of the tumor. It has been reported that early surgical treatment is effective in treating spontaneous rupture of hepatic angiomyolipoma with internal and peritoneal hemorrhage (9). In addition, HEAML tumors have low malignancy potential. Moreover, complete surgical resection can prevent local recurrence or metastasis (10). Approximately 10% of patients with HEAML who undergo surgical resection experience postoperative recurrence or metastasis (11). Postoperative radiotherapy and chemotherapy are ineffective, therefore, we suggest regular postoperative follow-up observation.

## 5. Conclusions

We present the case of a patient with HEAML and a history of breast cancer who presented with elevated AFP levels. Clinically, radiologically, and even pathologically, with special frozen pathological examination, HEAML can easily be misdiagnosed as other tumors. It is especially necessary to differentiate HEAML from hepatocellular carcinomas and metastatic tumors, necessitating the use of a specific IHC staining panel. Early surgical resection is the mainstay of treatment, and long-term postoperative follow-up is required.

## Patient perspective

All examination and treatment patients provided informed consent.

## Data Availability

The original contributions presented in the study are included in the article/Supplementary Material, further inquiries can be directed to the corresponding author/s.
